# Genome Editing in Cotton with the CRISPR/Cas9 System

**DOI:** 10.3389/fpls.2017.01364

**Published:** 2017-08-03

**Authors:** Wei Gao, Lu Long, Xinquan Tian, Fuchun Xu, Ji Liu, Prashant K. Singh, Jose R. Botella, Chunpeng Song

**Affiliations:** ^1^State Key Laboratory of Cotton Biology, Henan Key Laboratory of Plant Stress Biology, School of Life Sciences, Henan University Kaifeng, China; ^2^State Key Laboratory of Cotton Biology, Institute of Cotton Research of Chinese Academy of Agricultural Sciences Anyang, China; ^3^School of Agriculture and Food Sciences, University of Queensland, Brisbane QLD, Australia

**Keywords:** cotton, CRISPR/Cas9, deletion, genome editing, insertion, mutagenesis, transient transform

## Abstract

Genome editing is an important tool for gene functional studies as well as crop improvement. The recent development of the CRISPR/Cas9 system using single guide RNA molecules (sgRNAs) to direct precise double strand breaks in the genome has the potential to revolutionize agriculture. Unfortunately, not all sgRNAs are equally efficient and it is difficult to predict their efficiency by bioinformatics. In crops such as cotton (*Gossypium hirsutum* L.), with labor-intensive and lengthy transformation procedures, it is essential to minimize the risk of using an ineffective sgRNA that could result in the production of transgenic plants without the desired CRISPR-induced mutations. In this study, we have developed a fast and efficient method to validate the functionality of *sgRNAs* in cotton using a transient expression system. We have used this method to validate target sites for three different genes *GhPDS, GhCLA1*, and *GhEF1* and analyzed the nature of the CRISPR/Cas9-induced mutations. In our experiments, the most frequent type of mutations observed in cotton cotyledons were deletions (∼64%). We prove that the CRISPR/Cas9 system can effectively produce mutations in homeologous cotton genes, an important requisite in this allotetraploid crop. We also show that multiple gene targeting can be achieved in cotton with the simultaneous expression of several sgRNAs and have generated mutations in *GhPDS* and *GhEF1* at two target sites. Additionally, we have used the CRISPR/Cas9 system to produce targeted gene fragment deletions in the *GhPDS* locus. Finally, we obtained transgenic cotton plants containing CRISPR/Cas9-induced gene editing mutations in the *GhCLA1* gene. The mutation efficiency was very high, with 80.6% of the transgenic lines containing mutations in the *GhCLA1* target site resulting in an intense albino phenotype due to interference with chloroplast biogenesis.

## Introduction

The post-genomic era confronted researchers with the need to develop efficient tools for gene function studies (Zhang F. et al., 2014; Liu et al., 2015). Reverse genetics approaches such as gene silencing have been widely used by the scientific community to elucidate gene function and decipher regulatory mechanisms and metabolic pathways (Carroll, 2011; Jinek et al., 2012; Sun et al., 2016). However, some of the available gene silencing technologies such as RNA interference have a number of inherent shortcomings, such as stability and incomplete silencing that can complicate the interpretation of the resulting phenotypes (Boettcher and McManus, 2015; Xu et al., 2016). Targeted genome editing has a number of advantages over other approaches as it introduces mutations in the genome that are intrinsically stable and heritable over many generations (Hilscher et al., 2017).

Diverse approaches have been developed to accomplish target-specific genome editing, such as Zinc Finger Nucleases (ZFNs), Transcription Activator Like Effector Nucleases (TALENs), and Clustered Regularly Interspaced Short Palindromic Repeats (CRISPR)/CRISPR associated (Cas) protein systems (Gaj et al., 2013; Zhang et al., 2017). Despite their clear advantages over other approaches, ZFNs and TALENs were not widely adopted by the scientific community due to their technical complexity in the design and cloning of the molecular cassettes needed for the expression and targeting of the nuclease. The recent discovery of the CRISPR/Cas9 system has revolutionized the field as it uses a short RNA molecule to recognize the target site instead of the large polypeptides needed by ZFNs and TALENs. CRISPR/Cas9 has been rapidly adopted as the preferred genome editing tools and has been widely used in animal and plants due to its versatility, design simplicity, low cost and high efficiency (Shan et al., 2014; Wu et al., 2014; Bortesi and Fischer, 2015; Gao et al., 2015). The efficiency of sgRNAs directly affects the effective application of CRISPR/Cas9 in plants. Based on bioinformatics analysis, selection of targeted sites, and prediction of secondary structures were used to imporve the efficiency of sgRNAs (Ma et al., 2015). Protoplast transformation is also used for sgRNAs validation. However, protoplast isolation and transformation in some species are difficult to implement. There remains a need for a rapid, simple and efficient sgRNA selection method to develop CRISPR/Cas9 system.

Cotton is one of the most important fibers, a good source for biofuel production and an oil crop (Wang et al., 2012; Shi et al., 2014; Oliveira et al., 2016). Increased sequence availability has emphasized the need for rapid and cost-effective tools to create targeted mutations in order to perform much needed large-scale gene functional studies in cotton (Wang et al., 2010, 2012; Li et al., 2015; Zhang et al., 2015). CRISPR/Cas9 has been successfully used for gene editing in important crops and model systems such as rice, wheat, *Arabidopsis, Nicotiana*, Sorghum, poplar, maize, and tomato (Li et al., 2013; Nekrasov et al., 2013; Brooks et al., 2014; Fauser et al., 2014; Liang et al., 2014; Shan et al., 2014; Xu et al., 2014; Zhang H. et al., 2014; Fan et al., 2015; Mao et al., 2016). A growing number of agronomically useful genes are being identified in cotton, mostly involved in stress resistance and fiber development, and it is becoming urgent to develop a working CRISPR/Cas9 system for this crop (Jin and Liu, 2008; Taliercio et al., 2010; Gao et al., 2011, 2016b; Pan, 2013; Long et al., 2014). Recently, several reports have described the application of CRISPR/Cas9 in cotton targeting *MYB25, GFP, GhVP, GhCLA1*, or *GhARG* for genome editing, suggesting that CRISPR/Cas9 can be effectively used for cotton genome editing (Chen et al., 2017; Janga et al., 2017; Li C. et al., 2017; Wang P. et al., 2017; Wang Y. et al., 2017). However, the long and technically challenging transformation method for this crop limit the wide application of this technology.

In this study, we developed a fast method to experimentally validate *sgRNAs* for CRISPR/Cas9 gene editing in cotton using transient expression in cotyledon, which can be accomplished in 3 days. The new method was successfully used for multiple purposes, including validation of *sgRNAs* targeting individual genes (*GhPDS, GhCLA1*, and *GhEF1*), simultaneous editing of homeologous genes in the cotton polyploid genome and genomic fragment deletions. Moreover, CRISPR/Cas9-induced mutations were produced in stably transformed cotton plants targeting the *GhCLA1* resulting in typical albino phenotypes in the regenerated plants.

## Materials and Methods

### Plant Materials and Growth Conditions

Cotton (*Gossypium hirsutum* L. cv. ‘TM-1’) seeds were imbibed in deionized water for 8 h before being allowed to germinate at 28°C for 24 h in the dark. Following germination, the seedlings were grown in soil at 22/25°C (night/day) under 12 h/12 h light/dark conditions. Ten-day-old seedlings were used for transient transformation experiments. *G. hirsutum* L. cv. ‘YZ-1’ was used for stable transformation.

### Construction of CRISPR/Cas9 Vectors for Targeted Gene Editing

We used the pYLCRIPSR/Cas9 multiplex binary vector system containing a plant codon optimized Cas9 gene (Ma et al., 2015). To confirm target specificity in the genome of cotton during target site selection, a BLAST search was conducted using the cotton genome database^1^. The more detailed procedure was performed as previously described (Ma et al., 2015). Subsequently, the candidate *sgRNA* sequences were subjected to secondary structure analysis using an RNA folding platform^2^. Overlapping PCR was conducted to amplify the targeted sequences for the *sgRNAs*, including the two promoters utilized in this study (AtU6-29 and or AtU3b) using the primers listed in Supplementary Table S1. PCR products containing the targeted sequences were ligated into the CRISPR/Cas9 expression cassette via Golden Gate cloning, and more details were described in a previous report (Ma et al., 2015).

### Transient Expression and Stable Transformation

CRISPR/Cas9 vectors were transferred into *Agrobacterium tumefaciens* (GV3101 and LBA4404) for transient and stable transformation experiments, respectively. For transient transformation, GV3101 was infiltrated into cotyledons of 10-day-old cotton seedlings using a needless syringe. The seedlings were incubated in a plant growth chamber at 25°C. After incubation for 48 h, the infiltrated cotyledons were harvested for genomic DNA isolation and PCR/RE analysis (Gao et al., 2011). Stable transformation was performed in *G. hirsutum* L. cv. ‘YZ-1’ with LBA4404 as previously described (Jin et al., 2005).

### Detection of Genome Modifications

Genomic DNA was isolated from infiltrated cotyledons using a DNA extraction kit (Tiangen Biotech, Beijing). PCR was used to amplify a genomic fragment containing the target site and appropriate restriction enzymes used to digest PCR products in order to confirm mutations at the target site. The PCR amplicons were also cloned into a TA-cloning vector and sequenced (Sangon Biotech, Shanghai).

### RT-PCR Analysis

Total RNA was isolated from transgenic cotton lines using the Aidlab RNA extraction kit (Aidlab Biotechnologies, China). First-strand cDNA was performed from 1 μg of total RNA using the M-MLV Reverse Transcription System (Promega, United States). PCR was performed as follows: 95°C for 5 min, followed by 28 cycles of amplification (95°C for 10 s, 57°C for 30 s, and 72°C for 30 s), and a final incubation at 72°C for 7 min. The cotton gene *ubiquitin7* (*GhUB7*, Accession: DQ116441) was amplified as internal control.

## Results

### Strategy for Fast Target Validation in Cotton Cotyledons

**FIGURE 1:**
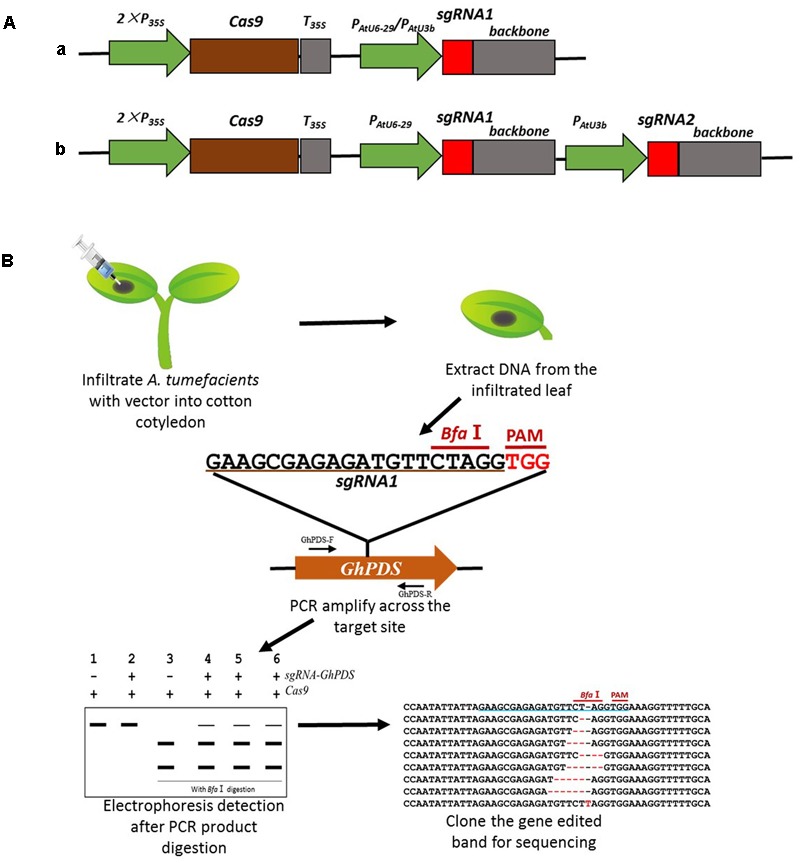
Strategy used for the generation of the CRISPR/Cas9 system for mutagenesis in cotton via transient expression system. **(A)** Schematic representation of CRISPR/Cas9 vectors containing one (a) or two (b) *sgRNA* expression cassettes, used for transient or stable transformation in cotton. **(B)** Schematic description of the transient transformation method and detection of mutations.

The available cotton transformation methods are technically demanding and time-consuming with an average of 10 months to produce T_0_ transgenic lines (Jin et al., 2005). Given the length and difficulty involved in obtaining stable cotton transgenic lines, it was critical to develop a fast method to test the efficiency of the *sgRNAs*. We therefore designed a fast *sgRNA* validation method for our research (**Figure 1**). Transient expression was achieved by infiltrating 10-day-old cotton cotyledons with *A*. *tumefaciens* harboring the appropriate CRISPR/Cas9 vectors (**Figure 1A**). The infiltrated cotyledons were harvested 48 h later, genomic DNA isolated and a fragment containing the target site amplified by PCR. Targeted sequences had been carefully selected to contain a restriction site in the vicinity of the PAM, therefore CRISPR-induced mutagenesis would likely destroy the enzyme recognition site. PCR amplification products were digested with the appropriate restriction enzyme and analyzed by gel electrophoresis to detect the loss of the restriction site. Finally, the gene-edited products, appearing in the electrophoresis as a high molecular size band were isolated and sequenced to re-confirm the modification and determine the nature of the CRISPR/Cas9-induced mutations (**Figure 1B**). This method proved to be very convenient to quickly validate the efficiency of the different *sgRNAs*, being capable to simultaneously test 4–6 different targets in less than 1 week using transient expression in cotton cotyledons.

### CRISPR/Cas9-Induced Mutations Using Transient Expression

**FIGURE 2:**
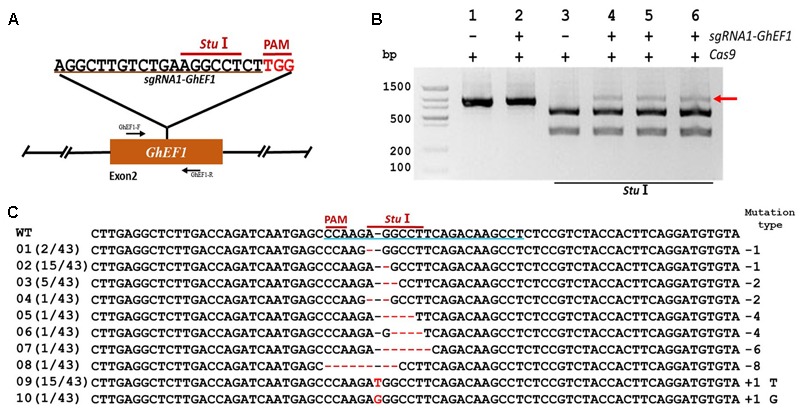
CRISPR/Cas9-mediated targeted mutagenesis of *GhEF1* in cotton cotyledons. **(A)** Target site of the *sgRNA1-GhEF1* used for the transient expression experiments and relative position in the *GhEF1* gene. GhEF1-F and GhEF1-R, forward and reverse primers used for amplification of the genomic fragment. **(B)** Detection of *sgRNA1-GhEF1* targeted mutations. The gel image shows PCR amplification products from genomic DNA samples extracted after transient expression of CRISPR constructs containing Cas9 and/or the *sgRNA1-GhEF1* expression cassettes. Lanes 1, 2: undigested PCR products; lanes 3–6: PCR products digested with *Stu*I. The red arrow shows the PCR products lacking the *Stu*I site (due to the presence of a mutation) that were subsequently purified, cloned, and analyzed by sequencing. **(C)** Sequencing of mutated PCR products. The target sequence (*GhEF1*) underlined in blue. Deletions are shown as red dashes; insertions are denoted with red letters. The frequency of each mutation is shown on the left and the mutation types on the right.

**FIGURE 3:**
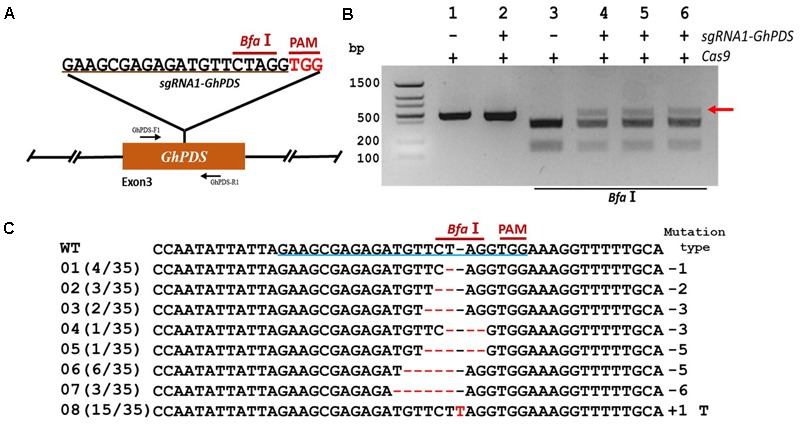
CRISPR/Cas9-mediated targeted mutagenesis of *GhPDS* in cotton cotyledons. **(A)** Target site of the *sgRNA1-GhPDS* used for the transient expression experiments and relative position in the *GhPDS* gene. GhPDS-F1 and GhPDS-R1, forward and reverse primers used for amplification of the genomic fragment. **(B)** Detection of *sgRNA1-GhPDS* targeted mutations. The gel image shows PCR amplification products from genomic DNA samples extracted after transient expression of CRISPR constructs containing Cas9 and/or the *sgRNA1-GhPDS* expression cassettes. Lanes 1, 2: undigested PCR products; lanes 3–6: PCR products digested with *Bfa*I. The red arrow shows the PCR products lacking the *Bfa*I site (due to the presence of a mutation) that were subsequently purified, cloned, and analyzed by sequencing. **(C)** Sequencing of mutated PCR products. The target sequence (*GhPDS*) underlined in blue. Deletions are shown as red dashes, insertions are denoted with red letters. The frequency of each mutation is shown on the left and the mutation types on the right.

In order to validate the transient transformation-based *sgRNA* validation method we choose the elongation factor-1 protein (GhEF1) as a target (D07G1160). GhEF1 catalyzes the binding of aminoacyl-tRNAs to the A-site of the ribosome during protein synthesis. The chosen 20-bp target sequence (*sgRNA1-GhEF1*) was located in exon #2 of the *GhEF1* gene and contained a *Stu*I restriction site 3 bases away from the PAM for detection of mutations (**Figure 2A**). Transient expression with binary vectors containing expression cassettes for Cas9 alone or together with *sgRNA1-GhEF1* produced PCR amplicons of approximately 0.87 kb in size (**Figure 2B**, lanes 1 and 2). As expected, digestion of the amplification product with *Stu*I produced two fragments of 554 and 314 bp in the absence of target sequence *sgRNA1-GhEF1* (**Figure 2B**, lane 3), while an extra, undigested fragment, was observed in the presence of such a sequence in the CRISPR/Cas9 cassette (**Figure 2B**, lanes 4–6). The undigested fragment was purified from the gel and cloned for sequence analysis. A total of 43 clones were analyzed with 63% of them harboring deletions (from 1 to 8 nucleotides in length), while 37% contained insertions (1 nucleotide) (**Figure 2C**). In total, 10 different mutations were detected, of which single base deletions (17/43) and single base insertions (15/43) were the most common types. Further validation of the transient transformation system was achieved using the phytoene desaturase (*PDS*) gene (D07G1160) (**Figures 3A,B**). In this case, 35 clones were sequenced revealing the presence of eight different mutation types, 57% of them deletions (from 1 to 6 nucleotides in length), and 43% insertions (1 nucleotide) (**Figure 3C**).

**FIGURE 4:**
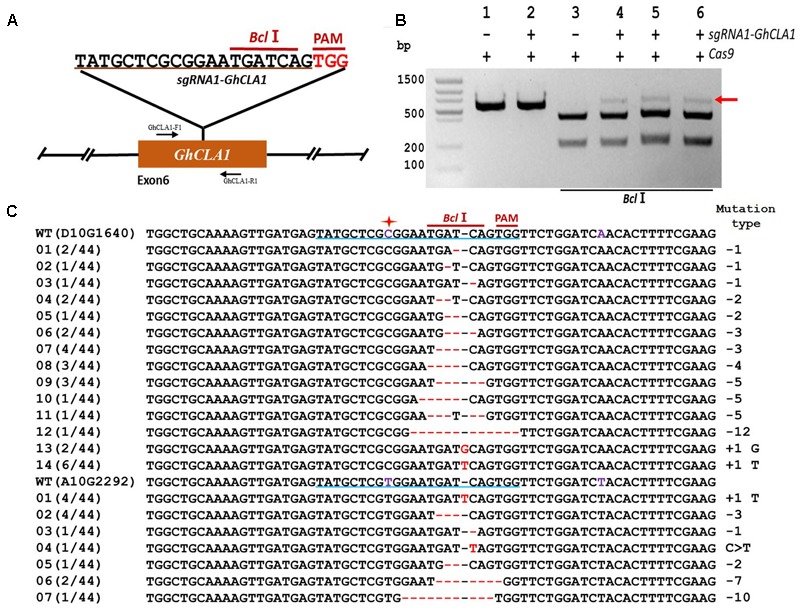
CRISPR/Cas9-mediated targeted mutagenesis of homeologous *GhCLA1* genes in cotton cotyledons. **(A)** Target site of the *sgRNA1-GhCLA1* used for the transient expression experiments and relative position in the homeologous *GhCLA1* genes (D10G1640 from D sub-genome and A10G229*2* from A sub-genome). GhCLA1-F1 and GhCLA1-R1, forward and reverse primers used for amplification of the genomic fragment. **(B)** Detection of *sgRNA1-GhCLA1* targeted mutations. The gel image shows PCR amplification products from genomic DNA samples extracted after transient expression of CRISPR constructs containing Cas9 and/or the *sgRNA1-GhCLA1* expression cassettes. Lanes 1, 2: undigested PCR products; lanes 3–6: PCR products digested with *Bcl*I. The red arrow shows the PCR products lacking the *Bcl*I site (due to the presence of a mutation) that were subsequently purified, cloned, and analyzed by sequencing. **(C)** Sequencing of mutated PCR products. The target sequence (*GhCLA1*) is underlined in blue. Deletions are shown as red dashes, insertions are denoted with red letters. The frequency of each mutation is shown on the left and the mutation types on the right.

The high number of homoeologous segments present in the genome of the allotetraploid cotton results in the existence of two or more copies for most genes (Wang et al., 2014; Gao et al., 2016a). In order to obtain a functional mutant in cotton, it is imperative to edit homoeologous sequences simultaneously, and in most cases, it is possible to find highly conserved CRISPR target sequences in the homeolog genes (Gao et al., 2016a). To test whether simultaneous mutation of homoeologous genes is feasible in cotton we chose Chloroplastos alterados 1 (*GhCLA1*) which is involved in chloroplast development and have two homoeologous sequences (D10G1640 from the sub-genome D and A10G2292 from the sub-genome A, **Figure 4A**). We designed a target sequence for D10G1640 that contained a single nucleotide difference at the 12th position upstream of the PAM site in A10G2292. The results of sequencing revealed successful editing events in D10G1640 as well as A10G2292, even with the imperfect target match, once more confirming the ability of CRISPR/Cas9 to edit homeologous genes in polyploids (Wang et al., 2014). As in the previous two experiments (**Figures 4B,C**), the frequency of deletion mutations was higher (70.45%) than insertions (27.17%).

Combined statistical analysis of our transient expression results indicates that the most common CRISPR/Cas9-induced mutations are deletions (69.93%) ranging from 1 to 6 nucleotides, while insertions accounted for (35.25%), all of them being single-nucleotide insertions (**Table 1**). Only one instance of nucleotide replacement was detected among the 122 mutations analyzed.

**Table 1:** Determination of mutation types of transient transformation with CRISPR/Cas9 vector in cotton cotyledon.

Mutation type	Description	No. of clones with mutations	Mutation rate	Total mutation rate
Deletion	1 bp	26	21.30%	63.93%
	2 bp	13	10.66%	
	3 bp	13	10.66%	
	4–6 bp	21	17.21%	
	>6 bp	5	4.10%	
Insertion	A/T	40	32.79%	35.25%
	C/G	3	2.46%	
Replacement	C > T	1	0.82%	0.82%

### CRISPR/Cas9-Mediated Mutation of Two Different Genes in Transient Expression Assays

**FIGURE 5:**
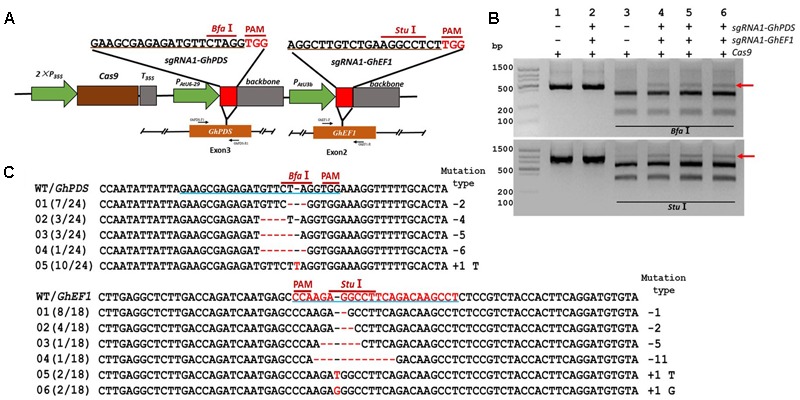
Simultaneous CRISPR/Cas9-mediated targeted mutagenesis of *GhPDS* and *GhEF1* genes in cotton cotyledons. **(A)** Target sites of the *sgRNA1-GhPDS* and *sgRNA1-GhEF1* used for the transient expression experiments and relative position in the GhPDS and *GhEF1* gene. GhPDS-F1, GhPDS-R1, GhEF1-F and GhEF1-R, forward and reverse primers used for amplification of the genomic fragments. **(B)** Detection of *sgRNA1-GhEF1* and *sgRNA1-GhEF1* targeted mutations. The gel images show PCR amplification products from genomic DNA samples extracted after transient expression of CRISPR constructs containing Cas9 and/or the *sgRNA1-GhPDS+sgRNA1-GhEF1* expression cassettes. Lanes 1, 2: undigested PCR products; lanes 3–6: PCR products digested with *Bfa*I (upper image) or *Stu*I (lower image). The red arrow shows the PCR products lacking the *Bfa*I site (upper image) or *Stu*I site (lower image) (due to the presence of a mutation) that were subsequently purified, cloned, and analyzed by sequencing. **(C)** Sequencing of mutated PCR products. The target sequences are underlined in blue. Deletions are shown as red dashes, insertions are denoted with red letters. The frequency of each mutation is shown on the left and the mutation types on the right.

CRISPR/Cas9 has been successfully used to create multiple mutations simultaneously (Zhang Y. et al., 2016), a feature that could prove extremely valuable for crops with lengthy and complicated transformation methods such as cotton. To validate the feasibility of creating double mutants, we used the previously described target sites for the *GhEF1* and *GhPDS* genes (**Figures 2, 3**) to build two *sgRNA* expression cassettes (*sgRNA1-GhEF1* and *sgRNA1-GhPDS*) and cloned both of them into a single CRISPR/Cas9 expression vector (**Figure 5A**). To minimize the probability of silencing due to the presence of repeated sequences in the transient expression cassette, expression of the *GhPDS* and *GhEF1 sgRNAs* was driven by the AtU6-29 and AtU3b promoters respectively. Transient expression of the dual *sgRNA* vector in cotton cotyledons induced mutations in both targeted genes as indicated by the presence of a ‘non-digested’ amplification product for both genes (*Bfa*I for *GhPDS* and *Stu*I for *GhEF1*) (**Figure 5B**). The undigested band was purified, cloned, and subjected to sequencing analysis (**Figure 5C**). The sequencing results showed the presence of multiple mutation types in both genes illustrating the suitability of the CRISPR/Cas9 system for the production of double/multiple mutants for functional genomics in cotton.

### CRISPR/Cas9-Mediated Gene Fragment Deletion in Cotton

**FIGURE 6:**
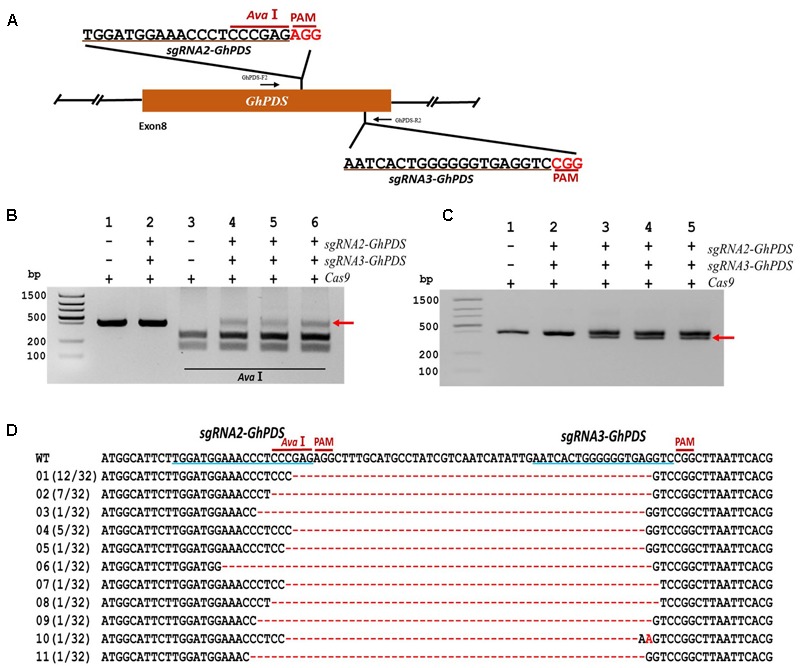
CRISPR/Cas9-mediated targeted gene deletion in cotton cotyledons. **(A)** Target sites of the *sgRNA2-GhPDS* and *sgRNA3-GhPDS* used for the transient expression experiments and relative position in the *GhPDS* gene. GhPDS-F2 and GhPDS-R2, forward and reverse primers used for amplification of the genomic fragment. **(B)** Detection of *sgRNA1-GhPDS* targeted mutations. The gel image shows PCR amplification products from genomic DNA samples extracted after transient expression of CRISPR constructs containing Cas9 and/or the *sgRNA2-GhPDS*+ *sgRNA3-GhPDS* expression cassettes. Lanes 1, 2: undigested PCR products; lanes 3–6: PCR products digested with *Ava*I. The red arrow shows the PCR products lacking the *Bfa*I site (due to the presence of a mutation) that were subsequently re-amplified. **(C)** Gel electrophoresis of PCr amplified products from **(B)**. The red arrow shows the smaller size PCR products (due to the presence of a deletion) that were subsequently purified, cloned, and analyzed by sequencing. **(D)** Sequencing of mutated PCR products from **(C)**. The target sequences are underlined in blue. Deletions are shown as red dashes, insertions are denoted with red letters. The frequency of each mutation is shown on the left.

Simultaneous targeting of two sites within the same gene can improve the mutagenesis efficiency of CRISPR/Cas9 and lead to the deletion of the gene fragment contained between the two targeted sites, thus increasing efficiency and providing an easier detection method (Zhang B. et al., 2016). To study whether CRISPR/Cas9 can be used for gene fragment deletion in cotton, two *sgRNAs* (*sgRNA2-GhPDS* and *sgRNA3-GhPDS*) were designed targeting sequences sites within the 8th exon of the *GhPDS* gene (48 bp apart) (**Figure 6A**). The target site of *sgRNA2-GhPDS* contained an *Ava*I restriction site for convenient detection of the mutation. Transient expression experiments were conducted with a binary vector containing both *sgRNA* as well as the Cas9 expression cassettes in cotton cotyledons. Genomic DNA was purified, and PCR performed using a pair of primers external to the targets to amplify a genomic fragment containing both target sites. Analysis of the PCR amplicons showed that co-expression of Cas9 and the two *sgRNAs* resulted in the appearance of a product non-digestible with *Ava*I (**Figure 6B**). The high molecular weight bands present in lanes 4, 5, and 6 of **Figure 6B** were purified and re-amplified by PCR revealing the existence of two molecular species with slightly different sizes (**Figure 6C**; lanes 3, 4, and 5). We hypothesized that the lower molecular size amplicons were produced by deletion of the genomic fragment between the two targeted sites and were therefore purified, cloned, and sequenced. Sequence analysis of 32 clones confirmed our hypothesis showing the existence of deletion ranging from 50 to 61 nucleotides between both target sites (**Figure 6D**).

### Gene Editing in Cotton by Stable Integration of CRISPR/Cas9 Cassettes

**FIGURE 7:**
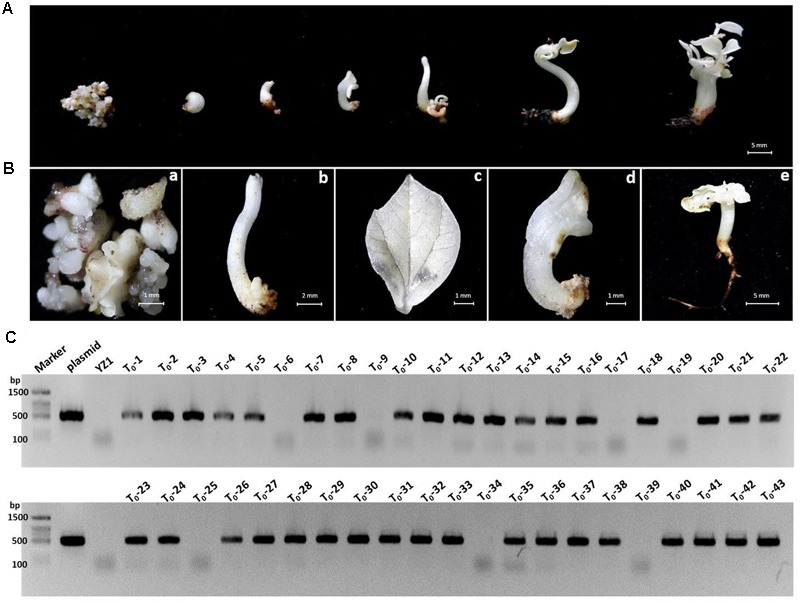
Phenotypes of transgenic cotton tissues carrying CRISPR/Cas9 cassettes targeting the *GhCLA1* gene. **(A)** The albino phenotype shown at different developmental stages after transformation. **(B)** Bleached phenotype of cotton calli (a), stem (b), leaf (c), malformed seedling (d), and normal seedling (e). **(C)** PCR analysis of genomic DNA isolated from 43 putative transgenic lines to detect the *Cas9* coding region. Lanes: Marker, molecular weight markers; Plasmid, positive control vector pYLCRISPR/Cas9-N; YZ-1, WT; T_0_-T_43_, independent kanamycin-resistant calli lines.

**FIGURE 8:**
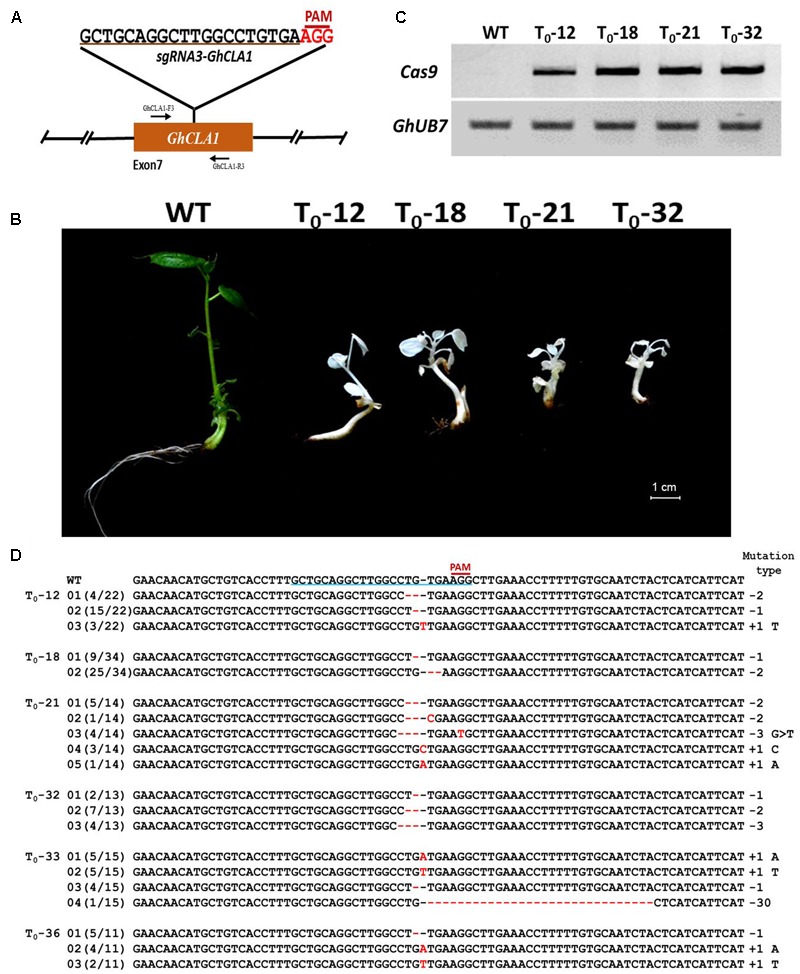
CRISPR/Cas9-mediated genome editing in transgenic cotton. **(A)** Target site of the *sgRNA3-GhCLA1* used for the trasnformation experiments and relative position in the *GhCLA1* gene. GhCLA-F3 and GhCLA-R3, forward and reverse primers used for amplification of the genomic fragment. **(B)** Phenotypes of cotton seedlings from wild-type plants and four T_0_ (12, 18, 21, and 32) transgenic cotton lines obtained via *Agrobacterium*-mediated stable transformation with a CRISPR/Cas9 construct targeting the *GhCLA1* gene. **(C)** Electrophoresis gel showing the result of RT-PCR from total RNA isolated from WT plants and four transgenic cotton lines. *GhUB7* expression was detected as internal control. **(D)** Targeted mutagenesis of *GhCLA1*. Sequencing of PCR products amplified from genomic regions containing the targeted site. Deletions are shown as red dashes, insertions are denoted with red letters. The target sequence is underlined in blue. The frequency of each mutation is shown on the left and the mutation types on the right.

Our developed transient transformation system was quite efficient in elucidating the suitability of CRISPR/Cas9 potential target sites in cotton cotyledons. However, this method does not produce stably mutated plants, and it is therefore not suitable for phenotypic studies. We used *A. tumefaciens*-mediated hypocotyl transformation to produce stable transgenic cotton lines and further confirm the feasibility of the CRISPR/Cas9 system in cotton. The *GhCLA1* was selected as a target given the easily observable photobleaching phenotype caused by its inactivation (Gao et al., 2011). Some of the calli regenerated on selection medium approximately 3 months after *Agrobacterium*-mediated transformation with the CRISPR/Cas9 binary construct showed an albino phenotype that was maintained during the entire regeneration process in stems, leaves and entire seedlings (**Figures 7A,B**). Ultimately, 43 putative transgenic T_0_ cotton lines were obtained, 36 of which tested positive for the Cas9 transgene by PCR (**Figure 7C** and Supplementary Figure S1). Two PCR primers flanking the *sgRNA3-GhCLA1* target site in the 7th exon of *GhCLA1* (**Figure 8A**) were used to amplify the genomic fragment in the transgenic lines. Sequence analysis of the amplification products revealed the presence of mutations in 29 of the 36 positive transgenic lines (80.56%) (Supplementary Note S1). Unfortunately, the severe phenotypic effects caused by the inactivation of GhCLA1 (inhibition of chloroplast biogenesis), hindered the regeneration process and only some of the initial transgenic calli were successfully regenerated into plantlets, all of which showed photobleaching and severely stunted growth (**Figure 8B**). RT-PCR analysis revealed the accumulation of Cas9 transcripts in albino mutants (**Figure 8C**). Six regenerated albino plantlets were analyzed for the presence of CRISPR/Cas9-induced mutations in the *GhCLA1* target site. All T_0_ plants showed mutations in the gene with deletions (78.90%) being more frequent than insertions (21.1%), in agreement with our observations from the transient expression experiments (**Figure 8D**). Silencing of *GhCLA1* using the tobacco rattle virus (TRV)-mediated virus-induced gene silencing (VIGS) also produced an albino phenotype, but this silencing method resulted in uneven and less intense photobleaching compared to the CRISPR/Cas9 mutated plants (Supplementary Figure S2), highlighting the superiority of the CRISPR/Cas9 system over RNAi-based silencing approaches.

## Discussion

Despite cotton’s global economic importance, only a few gene functional studies were reported (Gao et al., 2011; Li et al., 2015; Zhang et al., 2015). Due to the polypoid nature of the crop, many important agronomic and quality traits controlling fiber quality, yield or defense resistance are regulated by multiple genes or genes with multiple copies, making it difficult to perform gene functional studies. Thus, most genes in the cotton genome have no proven function, or their functional annotation has been inferred from homology to genes characterized in other plants. The advent of CRISPR/Cas9 has provided an invaluable tool for genetic studies in a large variety of plant species (Li et al., 2013; Nekrasov et al., 2013; Xu et al., 2014; Zhou et al., 2014; Fan et al., 2015; Sun et al., 2015). During the preparation of this manuscript, several reports have described the use of CRISPR/Cas9 in cotton (Chen et al., 2017; Janga et al., 2017; Li C. et al., 2017; Wang P. et al., 2017; Wang Y. et al., 2017).

The successful application of the CRISPR/Cas9 system for crop improvement or functional analyses relies on the generation of stably transformed mutants in order to perform phenotypic characterization of homozygous stable mutants. The sequence of the target site contained in the *sgRNA* is a key factor affecting the overall mutagenesis efficiency of the CRISPR/Cas9 system as different *sgRNAs* can result in very different efficiencies when targeting the same gene (Ma et al., 2016). The generation of cotton mutants utilizing stable transformation is a labor-intensive and time consuming process, and therefore it is essential to select the best possible *sgRNA* in order to reduce the workload. Most of the *sgRNA* design and selection process is currently based on bioinformatics analysis (Fan et al., 2015; Ma et al., 2016; Sun et al., 2016; Zhang B. et al., 2016). Even though bioinformatics analysis is essential to predict the specificity and theoretical efficiency of the target sites (Ma et al., 2015), our work provides a fast and effective method to experimentally validate candidate *sgRNAs*. Based on our transient expression results targeting three different genes (*GhEF1, GhPDS*, and *GhCLA1*), the most common CRISPR/Cas9-induced mutations were deletions, which is consistent with the results reported in *Arabidopsis*, tobacco, rice and other species (Nekrasov et al., 2013; Li et al., 2014; Xu et al., 2014; Zhou et al., 2014; Fan et al., 2015; Sun et al., 2015). It is worth noting that the *sgRNAs* validation process can be completed in 3 days using our transient transformation method.

Cotton is an allotetraploid derived from hybridization and polyploidization of the A and D diploid genomes which have a high content of repetitive DNA (Li et al., 2015; Zhang et al., 2015). Thus, to perform functional studies, it is necessary to mutate multiple homoeoalleles (Wang et al., 2014). In this research, we designed a *sgRNA* targeting both *GhCLA1* homeoalleles (D10G1640 and A10G2292) with a single-base difference in the target site and produced mutations in both alleles. In addition to single mutants, functional genomics research requires the production of double and multiple mutants in many occasions (Thung et al., 2012; Shi et al., 2016; Emonds-Alt et al., 2017; Li P. et al., 2017). Simultaneous editing of multiple genes using CRISPR/Cas9 has been reported in some plant species (Nekrasov et al., 2013; Xu et al., 2014; Fan et al., 2015; Sun et al., 2015). For example, a multiplex system targeting six of the 14 PYL families of ABA receptor genes was used in a single transformation experiment in *Arabidopsis* and homozygous sextuple mutants identified in the T3 progeny (Zhang Z. et al., 2016). We proved that simultaneous expression of two *sgRNAs* targeting *GhPDS* and *GhEF1* produced mutations in both genes in cotton cotyledons. CRISPR/Cas9 can also be used to produce genomic deletions between two simultaneously targeted sites in many plant species such as *Arabidopsis*, rice, tobacco and wheat with deletion efficiency being higher for short fragments than long fragments (Nekrasov et al., 2013; Fauser et al., 2014; Liang et al., 2014; Shan et al., 2014; Xing et al., 2014; Ma et al., 2015; Xie et al., 2015). In our work, deletion of 50–61 bp fragments between two target sites were detected, indicating that CRISPR/Cas9 can be applied for the deletion of chromosomal fragments in cotton.

Stably transformed cotton plants are needed for most practical applications of the CRISPR/Cas9 system, especially when studying agronomically important traits. We produced 29 T_0_ independent transgenic lines with CRISPR/Cas9 constructs targeting *GhCLA1* with gene editing events detected in ∼80% of them. This mutation frequency was similar to those previous reports in other plants despite the large size of the cotton genome (Ma et al., 2016; Zhang Y. et al., 2016). It is worth noting that the efficiency of CRISPR/Cas9 using stable transformation is very high (∼80%) compared to the transient transformation. It is due to the process of stable transformation which experienced a long time tissue culture process with kanamycin resistant screening. Our data indicate that genome size does not have a significant influence on the efficiency of targeted genome mutagenesis mediated by CRISPR/Cas9 system, contrasting with the suggestion that species-specific differences in NHEJ contribute significantly to the evolution of genome size (Puchta, 2005). CRISPR/Cas9-induced mutations in *GhCLA1* produced a more obvious and uniform albino phenotype than the RNA interference induced by VIGS, emphasizing the advantage of genomic mutations over RNAi for gene silencing. The site-specific mutations created by CRISPR/Cas9 occur mostly in somatic cells and can be accurately inherited in multiple generations in *Arabidopsis* and rice (Ma et al., 2015). Unfortunately, the extreme phenotypic effects caused by a mutation in *GhCLA1* precludes the possibility of establishing the inheritance patterns, and therefore, the heritability of CRISPR/Cas9-induced gene modifications in cotton requires further study.

In summary, our transient transformation system allows the fast validation of CRISPR/Cas9 sgRNA targets and could facilitate the adoption of high throughput functional genomic studies in cotton, especially in combination with the newly emerging viral-based CRISPR methods (Wang M. et al., 2017).

## Author Contributions

WG and JB analyzed and interpreted data and wrote the manuscript. LL performed the cotton stable transformation. XT performed the transient transformation analysis. FX, JL, and PS helped to reproduce cotton material. CS designed the study and supervised all of work. All authors read and approved the final manuscript.

## Conflict of Interest Statement

The authors declare that the research was conducted in the absence of any commercial or financial relationships that could be construed as a potential conflict of interest.

## Footnotes

1 http://mascotton.njau.edu.cn/html/Data/Genomefhsequence/1.html

2 http://unafold.rna.albany.edu/?q=mfold/RNA-Folding-Form2.3
