# Supplementary material for: Genome Editing in Cotton with the CRISPR/Cas9 System
Source: Front Plant Sci. 2017 Aug 3;8:1364. doi: 10.3389/fpls.2017.01364 (PMC5541054; doi:10.3389/fpls.2017.01364)
Supplement: Supplementary file 1 [file Data_Sheet_1.PDF]

Supplementary Material

Supplementary Table S1 List of primers used in this study.

Primer ID	primer sequence (5'-3')	Description
GhCLA1-gRT1+	TATGCTCGCGGAATGATCAGGTTTTAGAGCTAGAAAT	For *sgRNA1-GhCLA1* cloning
GhCLA1-U6-29T1-	CTGATCATTCCGCGAGCATACAATCTCTTAGTCGACT	
GhCLA1-gRT2+	CATGCAAAGGGCATATGACCGTTTTAGAGCTAGAAAT	For *sgRNA2-GhCLA1* cloning
GhCLA1-U3bT2-	GGTCATATGCCCTTTGCATGTGACCAATGTTGCTCC	
GhCLA1-gRT3+	CTGCAGGCTTGGCCTGTGAGTTTTAGAGCTAGAAAT	For *sgRNA3-GhCLA1* cloning
GhCLA1-U6-29T3-	TCACAGGCCAAGCCTGCAGCAATCTCTTAGTCGACT	
GhEF1-gRT1+	GGCTTGTCTGAAGGCCTCTGTTTTAGAGCTAGAAAT	For *sgRNA1-GhEF1* cloning
GhEF1-U3bT1-	AGAGGCCTTCAGACAAGCCTGACCAATGTTGCTCC	
GhPDS-gRT1+	AAGCGAGAGATGTTCTAGGGTTTTAGAGCTAGAAAT	For *sgRNA1-GhPDS* cloning
GhPDS-U6-29T1-	CCTAGAACATCTCTCGCTTCAATCTCTTAGTCGACT	
GhPDS-gRT2+	TGGATGGAAACCCTCCCGAGGTTTTAGAGCTAGAAAT	For *sgRNA2-GhPDS* cloning
GhPDS-U6-29T2-	CTCGGGAGGGTTTCCATCCACAATCTCTTAGTCGACT	
GhPDS-gRT3+	ATCACTGGGGGGTGAGGTCGTTTTAGAGCTAGAAAT	For *sgRNA3-GhPDS* cloning
GhPDS-U3b-T3-	GACCTCACCCCCCAGTGATTGACCAATGTTGCTCC	
GhPDS-F1	TGCATGATCCATCACTCAAGTTT	For mutant detection of *GhPDS*
GhPDS-R1	GAACGAAAGGCCCTTTCTTTC	

GhPDS-F2	ATTCATTTCGTGTTCATTCATTTGTAT	For mutant detection of *GhPDS*
GhPDS-R2	GAAAGTTCAACATCCATCAGCTAT	
GhCLA1-F1	GGATCTGAAAGGTGAAAGGAATC	For mutant detection of *GhCLA1*
GhCLA1-R1	TACCGTGATACTTGTCAGCAGCT	
GhCLA1-F2	TTTACTGGTGCCTCGATATCTGA	For mutant detection of *GhCLA1*
GhCLA1-R2	CATCGTGTACGACCTGTTGCAG	
GhCLA1-F3	CACGGTAACATACAGAATAAGCC	For mutant detection of *GhCLA1*
GhCLA1-R3	ACGTCATGTACGACCTGTTGC	
GhEF1-F	TGGTATCACCATTGATATTGCCT	For mutant detection of *GhEF1*
GhEF1-R	ATGACCTGAGAAGTGAAGTTTGC	
NPTⅡ.-F	GGCACAACAGACAATCGGC	For mutant detection of *NPTⅡ*
NPTⅡ-R	CGTAAAGCACGAGGAAGCG	
Cas9-RT-F	TCAACGTACATATCCCTACCG	For RT-PCR analysis
Cas9-RT-R	AGGCTCAAGACTTACGCTCAT	

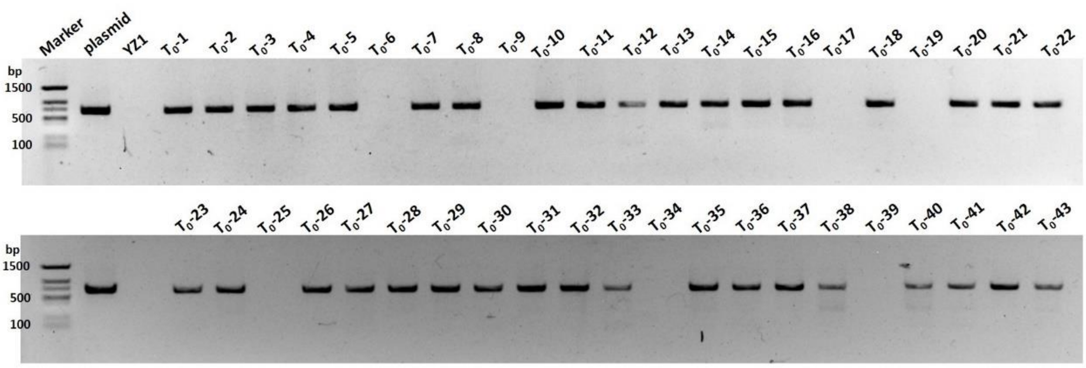

Supplementary Figure S1 PCR analysis of genomic DNA (43 transgenic lines) to detect the plant-selectable marker gene *NPT Ⅱ*. Lanes: Marker, molecular weight markers; Plasmid, positive control vector pYLCRISPR/Cas9-N; YZ1, genomic DNA of wild type plant; T_0-T_{43}, independent transformed kanamycin-resistant calluses lines.

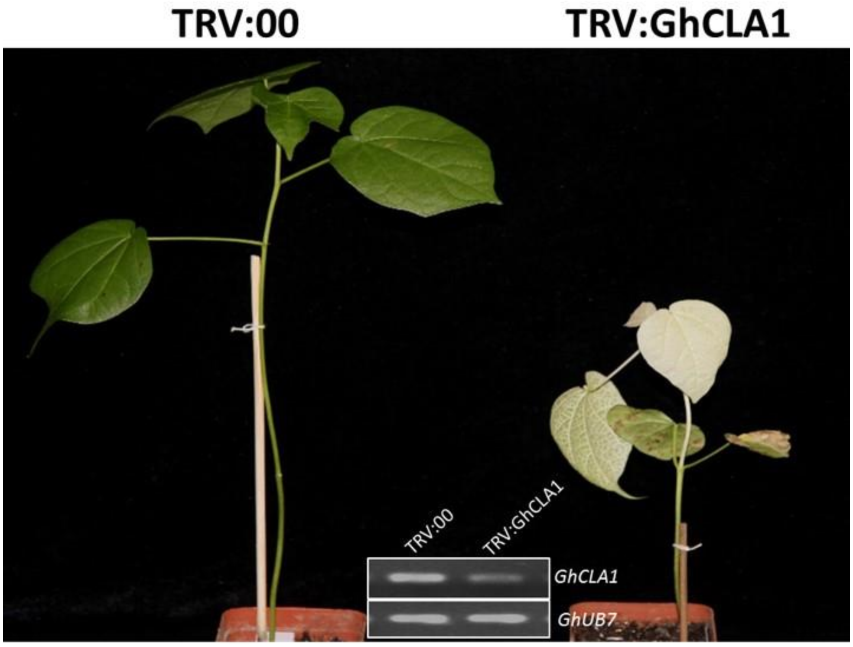

Supplementary Figure S2 Silencing of *GhCLA1* in cotton by tobacco rattle virus (TRV)-mediated virus-induced gene silencing (VIGS). Ten-day-old seedlings were used for infiltration with *A. tumefaciens* carrying *TRV:CLA1* vector. The photobleaching phenotype was appeared after two weeks inoculation. RT-PCR analysis indicated the expression of *CLA1* was reduced after inoculation.

Supplementary Note S1 Sequencing information of *GhCLA1* transgenic lines using CRISPR/Cas9 induced stable transformation.

```
Line-1
WT         GAACAACATGCTGTCACCTTTGCTGCAGGCTTGGCCTGT-GAAGGCTTGAAACCTTTTTGTGCAATCTACTCATCATTCAT
01(8/15)   GAACAACATGCTGTCACCTTTGCTGCAGGCTTGGCCTGTTGAAGGCTTGAAACCTTTTTGTGCAATCTACTCATCATTCAT +1
02(3/15)   GAACAACATGCTGTCACCTTTGCTGCAGGCTTGGCCT-T-GAAGGCTTGAAACCTTTTTGTGCAATCTACTCATCATTCAT -1
03(4/15)   GAACAACATGCTGTCACCTTTGCTGCAGGCTTGGCC--T-GAAGGCTTGAAACCTTTTTGTGCAATCTACTCATCATTCAT -2
Line-2
WT         GAACAACATGCTGTCACCTTTGCTGCAGGCTTGGCCTGT-GAAGGCTTGAAACCTTTTTGTGCAATCTACTCATCATTCAT
01(3/22)   GAACAACATGCTGTCACCTTTGCTGCAGGCTTGGCCT-T-GAAGGCTTGAAACCTTTTTGTGCAATCTACTCATCATTCAT -1
02(10/22)  GAACAACATGCTGTCACCTTTGCTGCAGGCTTGGCC--T-GAAGGCTTGAAACCTTTTTGTGCAATCTACTCATCATTCAT -2
03(9/22)   GAACAACATGCTGTCACCTTTGCTGCAGGCTTGGCCTGTTGAAGGCTTGAAACCTTTTTGTGCAATCTACTCATCATTCAT +1
Line-3
WT         GAACAACATGCTGTCACCTTTGCTGCAGGCTTGGCCTGTGAAGGCTTGAAACCTTTTTGTGCAATCTACTCATCATTCAT
01(3/9)    GAACAACATGCTGTCACCTTTGCTGCAGGCTTGGCC--TGAAGGCTTGAAACCTTTTTGTGCAATCTACTCATCATTCAT -2
02(4/9)    GAACAACATGCTGTCACCTTTGCTGCAGGCTTGGCCTGTGAAGGCTTGAAACCTTTTTGTGCAATCTACTCATCATTCAT 0
03(2/9)    GAACAACATGCTGTCACCTTTGCTGCAGGCTTGGCCT-TGAAGGCTTGAAACCTTTTTGTGCAATCTACTCATCATTCAT -1
Line-5
WT         GAACAACATGCTGTCACCTTTGCTGCAGGCTTGGCCTG-TGAAGGCTTGAAACCTTTTTGTGCAATCTACTCATCATTCAT
01(7/17)   GAACAACATGCTGTCACCTTTGCTGCAGGCTTGGCCTG-TGAAGGCTTGAAACCTTTTTGTGCAATCTACTCATCATTCAT 0
02(6/17)   GAACAACATGCTGTCACCTTTGCTGCAGGCTTGGCCT--TGAAGGCTTGAAACCTTTTTGTGCAATCTACTCATCATTCAT -1
03(4/17)   GAACAACATGCTGTCACCTTTGCTGCAGGCTTGGCCTGATGAAGGCTTGAAACCTTTTTGTGCAATCTACTCATCATTCAT +1
Line-7
WT         GAACAACATGCTGTCACCTTTGCTGCAGGCTTGGCCTG-TGAAGGCTTGAAACCTTTTTGTGCAATCTACTCATCATTCAT
01(10/13)  GAACAACATGCTGTCACCTTTGCTGCAGGCTTGGCCT--TGAAGGCTTGAAACCTTTTTGTGCAATCTACTCATCATTCAT -1
02(3/13)   GAACAACATGCTGTCACCTTTGCTGCAGGCTTGGCCTGTTGAAGGCTTGAAACCTTTTTGTGCAATCTACTCATCATTCAT +1
Line-8
WT         GAACAACATGCTGTCACCTTTGCTGCAGGCTTGGCCTGTGAAGGCTTGAAACCTTTTTGTGCAATCTACTCATCATTCAT
01(4/17)   GAACAACATGCTGTCACCTTTGCTGCAGGCTTGGCC--TGAAGGCTTGAAACCTTTTTGTGCAATCTACTCATCATTCAT -2
02(13/17)  GAACAACATGCTGTCACCTTTGCTGCAGGCTTGGCCTGTGAAGGCTTGAAACCTTTTTGTGCAATCTACTCATCATTCAT 0
Line-10
WT         GAACAACATGCTGTCACCTTTGCTGCAGGCTTGGCCTGTGAAGGCTTGAAACCTTTTTGTGCAATCTACTCATCATTCAT
01(1/17)   GAACAACATGCTGTCACCTTTGCTGCAGGCTTGGTCT-TGAAGGCTTGAAACCTTTTTGTGCAATCTACTCATCATTCAT -1
02(16/17)  GAACAACATGCTGTCACCTTTGCTGCAGGCTTGGCCTGTGAAGGCTTGAAACCTTTTTGTGCAATCTACTCATCATTCAT 0
Line-13
WT         GAACAACATGCTGTCACCTTTGCTGCAGGCTTGGCCTGTGAAGGCTTGAAACCTTTTTGTGCAATCTACTCATCATTCAT
01(7/15)   GAACAACATGCTGTCACCTTTGCTGCAGGCTTGGCCTGTGAAGGCTTGAAACCTTTTTGTGCAATCTACTCATCATTCAT 0
02(4/15)   GAACAACATGCTGTCACCTTTGCTGCAGGCTTGGCCTG-GAAGGCTTGAAACCTTTTTGTGCAATCTACTCATCATTCAT -1
03(4/15)   GAACAACATGCTGTCACCTTTGCTGCAGGCTTGGCCT-TGAAGGCTTGAAACCTTTTTGTGCAATCTACTCATCATTCAT -1

Line-14
WT         GAACAACATGCTGTCACCTTTGCTGCAGGCTTGGCCTGTGAAGGCTTGAAACCTTTTTGTGCAATCTACTCATCATTCAT
01(1/16)   GAACAACATGCTGTCACCTTTGCTGCAGGCTTGGCC--TG-AGGCTTGAAACCTTTTTGTGCAATCTACTCATCATTCAT -3
02(8/16)   GAACAACATGCTGTCACCTTTGCTGCAGGCTTGGCC--TGAAGGCTTGAAACCTTTTTGTGCAATCTACTCATCATTCAT -2
03(7/16)   GAACAACATGCTGTCACCTTTGCTGCAGGCTTGGCCT-TGAAGGCTTGAAACCTTTTTGTGCAATCTACTCATCATTCAT -1
Line-16
WT         GAACAACATGCTGTCACCTTTGCTGCAGGCTTGGCCTGT-GAAGGCTTGAAACCTTTTTGTGCAATCTACTCATCATTCAT
01(5/16)   GAACAACATGCTGTCACCTTTGCTGCAGGCTTGGCCTG---AAGGCTTGAAACCTTTTTGTGCAATCTACTCATCATTCAT -2
02(11/16)  GAACAACATGCTGTCACCTTTGCTGCAGGCTTGGCCTGTTGAAGGCTTGAAACCTTTTTGTGCAATCTACTCATCATTCAT +1
Line-20
WT         GAACAACATGCTGTCACCTTTGCTGCAGGCTTGGCCTGTGAAGGCTTGAAACCTTTTTGTGCAATCTACTCATCATTCAT
01(14/16)  GAACAACATGCTGTCACCTTTGCTGCAGGCTTGGCCT-TGAAGGCTTGAAACCTTTTTGTGCAATCTACTCATCATTCAT -1
02(2/16)   GAACAACATGCTGTCACCTTTGCTGCAGGCTTGGCC--TGAAGGCTTGAAACCTTTTTGTGCAATCTACTCATCATTCAT -2
Line-22
WT         GAACAACATGCTGTCACCTTTGCTGCAGGCTTGGCCTGTGAAGGCTTGAAACCTTTTTGTGCAATCTACTCATCATTCAT
01(16/16)  GAACAACATGCTGTCACCTTTGCTGCAGGCTTGGCCT-TGAAGGCTTGAAACCTTTTTGTGCAATCTACTCATCATTCAT -1
Line-26
WT         GAACAACATGCTGTCACCTTTGCTGCAGGCTTGGCCTGT-GAAGGCTTGAAACCTTTTTGTGCAATCTACTCATCATTCAT
01(4/11)   GAACAACATGCTGTCACCTTTGCTGCAGGCTTGGCCTGT-GAAGGCTTGAAACCTTTTTGTGCAATCTACTCATCATTCAT 0
02(4/11)   GAACAACATGCTGTCACCTTTGCTGCAGGCTTGGCC--T-GAAGGCTTGAAACCTTTTTGTGCAATCTACTCATCATTCAT -2
03(1/11)   GAACAACATGCTGTCACCTTTGCTGCAGGCTTGGCCTGTTGAAGGCTTGAAACCTTTTTGTGCAATCTACTCATCATTCAT +1
04(1/11)   GAACAACATGCTGTCACCTTTGCTGCAGGCTTGGCCT-T-GAAGGCTTGAAACCTTTTTGTGCAATCTACTCATCATTCAT -1
05(1/11)   GAACAACATGCTGTCACCTTTGCTGCAGGCTTGGCCTG-----GCCTTCAAACCTTTTTGTGCAATCTACTCATCATTCAT -4
Line-27
WT         GAACAACATGCTGTCACCTTTGCTGCAGGCTTGGCCTGT-GAAGGCTTGAAACCTTTTTGTGCAATCTACTCATCATTCAT
01(18/20)  GAACAACATGCTGTCACCTTTGCTGCAGGCTTGGCCTGT-GAAGGCTTGAAACCTTTTTGTGCAATCTACTCATCATTCAT 0
02(2/20)   GAACAACATGCTGTCACCTTTGCTGCAGGCTTGGCCTGTTGAAGGCTTGAAACCTTTTTGTGCAATCTACTCATCATTCAT +1
Line-28
WT         GAACAACATGCTGTCACCTTTGCTGCAGGCTTGGCCTGTGAAGGCTTGAAACCTTTTTGTGCAATCTACTCATCATTCAT
01(8/14)   GAACAACATGCTGTCACCTTTGCTGCAGGCTTGGCCT--GAAGGCTTGAAACCTTTTTGTGCAATCTACTCATCATTCAT -2
02(4/14)   GAACAACATGCTGTCACCTTTGCTGCAGGCTTGGCCT-TGAAGGCTTGAAACCTTTTTGTGCAATCTACTCATCATTCAT -1
03(2/14)   GAACAACATGCTGTCACCTTTGCTGCAGGCTTGGCCTGTGAAGGCTTGAAACCTTTTTGTGCAATCTACTCATCATTCAT 0
Line-30
WT         GAACAACATGCTGTCACCTTTGCTGCAGGCTTGGCCTG-TGAAGGCTTGAAACCTTTTTGTGCAATCTACTCATCATTCAT
01(2/15)   GAACAACATGCTGTCACCTTTGCTGCAGGCTTGGCCTGTTGAAGGCTTGAAACCTTTTTGTGCAATCTACTCATCATTCAT +1
02(9/15)   GAACAACATGCTGTCACCTTTGCTGCAGGCTTGGCC---TGAAGGCTTGAAACCTTTTTGTGCAATCTACTCATCATTCAT -2
02(4/15)   GAACAACATGCTGTCACCTTTGCTGCAGGCTTGGCCT--TGAAGGCTTGAAACCTTTTTGTGCAATCTACTCATCATTCAT -1
```

```
Line-31
WT        GAACAACATGCTGTCACCTTTGCTGCAGGCTTGGCCTGTGAAGGCTTGAAACCTTTTTGTGCAATCTACTCATCATTCAT
01(5/16)  GAACAACATGCTGTCACCTTTGCTGCAGGCTTGGCCT-TGAAGGCTTGAAACCTTTTTGTGCAATCTACTCATCATTCAT -1
02(11/16) GAACAACATGCTGTCACCTTTGCTGCAGGCTTGGCCTGTGAAGGCTTGAAACCTTTTTGTGCAATCTACTCATCATTCAT  0
Line-35
WT        GAACAACATGCTGTCACCTTTGCTGCAGGCTTGGCCTG-TGAAGGCTTGAAACCTTTTTGTGCAATCTACTCATCATTCAT
01(8/11)  GAACAACATGCTGTCACCTTTGCTGCAGGCTTGGCCT--TGAAGGCTTGAAACCTTTTTGTGCAATCTACTCATCATTCAT -1
02(1/11)  GAACAACATGCTGTCACCTTTGCTGCAGGCT-------------------------------------ACTCATCATTCAT -36
03(2/11)  GAACAACATGCTGTCACCTTTGCTGCAGGCTTGGCCTGATGAAGGCTTGAAACCTTTTTGTGCAATCTACTCATCATTCAT +1
Line-38
WT        GAACAACATGCTGTCACCTTTGCTGCAGGCTTGGCCTGT-GAAGGCTTGAAACCTTTTTGTGCAATCTACTCATCATTCAT
01(9/13)  GAACAACATGCTGTCACCTTTGCTGCAGGCTTGGCCTGTTGAAGGCTTGAAACCTTTTTGTGCAATCTACTCATCATTCAT +1
02(3/13)  GAACAACATGCTGTCACCTTTGCTGCAGGCTTGGCC--T-GAAGGCTTGAAACCTTTTTGTGCAATCTACTCATCATTCAT -2
02(1/13)  GAACAACATGCTGTCACCTTTGCTGCAGGCTTGGCCT-T-GAAGGCTTGAAACCTTTTTGTGCAATCTACTCATCATTCAT -1
Line-40
WT        GAACAACATGCTGTCACCTTTGCTGCAGGCTTGGCCTG-TGAAGGCTTGAAACCTTTTTGTGCAATCTACTCATCATTCAT
01(6/10)  GAACAACATGCTGTCACCTTTGCTGCAGGCTTGGCCTGTTGAAGGCTTGAAACCTTTTTGTGCAATCTACTCATCATTCAT +1
02(3/10)  GAACAACATGCTGTCACCTTTGCTGCAGGCTTGGCCT--TGAAGGCTTGAAACCTTTTTGTGCAATCTACTCATCATTCAT -1
02(1/10)  GAACAACATGCTGTCACCTTTGCTGCAGGCTTGGCC---TGAAGGCTTGAAACCTTTTTGTGCAATCTACTCATCATTCAT -2
Line-41
WT        GAACAACATGCTGTCACCTTTGCTGCAGGCTTGGCCTGTGAAGGCTTGAAACCTTTTTGTGCAATCTACTCATCATTCAT
01(17/17) GAACAACATGCTGTCACCTTTGCTGCAGGCTTGGCCT-TGAAGGCTTGAAACCTTTTTGTGCAATCTACTCATCATTCAT -1
Line-42
WT        GAACAACATGCTGTCACCTTTGCTGCAGGCTTGGCCTGTGAAGGCTTGAAACCTTTTTGTGCAATCTACTCATCATTCAT
01(12/16) GAACAACATGCTGTCACCTTTGCTGCAGGCTTGGCCT-TGAAGGCTTGAAACCTTTTTGTGCAATCTACTCATCATTCAT -1
02(4/16)  GAACAACATGCTGTCACCTTTGCTGCAGGCTTGGCC--TGAAGGCTTGAAACCTTTTTGTGCAATCTACTCATCATTCAT -2
Line-43
WT        GAACAACATGCTGTCACCTTTGCTGCAGGCTTGGCCTGTGAAGGCTTGAAACCTTTTTGTGCAATCTACTCATCATTCAT
01(2/17)  GAACAACATGCTGTCACCTTTGCTGCAGGCTTGGCCT-TGAAGGCTTGAAACCTTTTTGTGCAATCTACTCATCATTCAT -1
02(4/17)  GAACAACATGCTGTCACCTTTGCTGCAGGCTTGGCCTGTGAAGGCTTGAAACCTTTTTGTGCAATCTACTCATCATTCAT  0
03(11/17) GAACAACATGCTGTCACCTTTGCTGCAGGCTTGGCCT--GAAGGCTTGAAACCTTTTTGTGCAATCTACTCATCATTCAT -2
```
